# Routine Histological Examination of Prepuce in Pathological Phimosis: A Recommended Sustainable Practice

**DOI:** 10.7759/cureus.86653

**Published:** 2025-06-24

**Authors:** Rajkiran S Raju, Ankita Sharma, Kiran Mahadevappa, Ishwara Bhat, Inchara Y Kalegowda, Prasanna Kumar

**Affiliations:** 1 Pediatric Surgery, St John's Medical College Hospital, Bangalore, IND; 2 Dermatology, St John's Medical College Hospital, Bangalore, IND; 3 Pathology, St John's Medical College Hospital, Bangalore, IND

**Keywords:** biopsy, circumcision, lichen sclerosus, pathological phimosis, topical steroid

## Abstract

Background

Phimosis is a frequent indication for pediatric outpatient referral. Pathological phimosis results from chronic inflammatory changes, often with lichenoid changes resulting in a hypopigmented indurated preputial plaque. Treatment of preputial lichen sclerosus is circumcision followed by long-term topical steroid application in histologically confirmed cases. Routine histopathologic examination of circumcision specimens is advisable to detect early cases of lichen sclerosus.

Materials and methods

A retrospective chart review of children operated for pathological phimosis over a 32-month period was conducted. Demographic details, clinical symptomatology, histological findings, and outcomes at follow-up were collated and analyzed.

Results

Of the 30 patients enrolled in this study, the mean age of presentation was 9.6 years. Inability to retract the foreskin was seen in all patients, and preputial ballooning were seen in 20 (67%) patients. Biopsy was available in 25 patients, with all specimens demonstrating abnormal findings: lichen sclerosus (18; 72%), lichenoid dermatitis (4; 16%), and chronic non-specific inflammation (3; 12%). Meatal involvement was seen in four patients, with all improved with prolonged topical steroid therapy.

Conclusion

Recurrence of phimosis or failure despite topical steroid therapy is likely to represent pathological phimosis. Routine biopsy of all pathological phimosis specimens is recommended. Changes in interface dermatitis and lichenoid lymphocytic inflammation are the commonest findings on biopsy, and long-term steroids and regular follow-up are imperative.

## Introduction

Circumcision is a commonly performed office procedure worldwide, with the United States of America, Central Eastern, and Eastern Africa reporting circumcision rates of 70-90%; the reported estimates from the Indian Subcontinent are 13-15%. [1]. Although ritual circumcision is the commonest indication, circumcision is performed for urological indications also. Phimosis denotes the inability to retract the prepuce, leading to the narrowing of the preputial inlet for voiding urine. In children, phimosis is mostly physiological in the first five years of life, with the preputial skin being redundant yet supple, commonly referred to as “preputial adhesions” [2]. Pathological phimosis (PP) refers to an abnormally tight prepuce resulting from secondary skin changes occurring in the preputial skin. Balanitis xerotica obliterans (BXO), often used synonymously with lichen sclerosus, is a chronic mucocutaneous inflammatory process of unknown etiology with secondary sclerosis affecting the inner prepuce, coronal sulcus, and mucosal glans [3]. The management of most phimosis including physiological phimosis is a short course (three weeks) of topical steroid preparation application, followed by preputial adhesiolysis [4]. However, refractory phimosis (unresponsive or recurrent) is usually a harbinger of BXO. Treatment is circumcision followed by a long course (six months in proven cases) of topical steroids. If untreated, these lesions progress to catastrophic urethral meatal strictures with penile involvement [5]. These are potentially premalignant, and malignant transformation is rarely reported [6]. The need for routine histological review of pathological prepuce at circumcision stemmed from observation of a high incidence of lichenoid changes on routine histological examination of circumcised prepuce specimens, with BXO being underestimated in 30-50% of the cases. There are no guidelines or prescribed practices recommending routine histological examination of prepuce at circumcision for PP/BXO [7]. This study aims to highlight the importance of routine histological examination of the prepuce for BXO/PP in children.

## Materials and methods

We performed a retrospective chart review of children (0-18 years) admitted to the Department of Pediatric Surgery from January 2022 to August 2024. Clinical data included demographic profile, symptomatology, and follow-up findings at six months. Slides and blocks of the pertaining cases were retrieved from the archives of the Department of Pathology and reviewed for histopathologic features. Findings were collated and analyzed. Mean and range were used to demonstrate the age distribution, and descriptive statistics were used to demonstrate the distribution pattern of symptomatology and types of histological appearance at biopsy.

Inclusion and exclusion criteria

Children admitted with the diagnosis of PP (clinical features of BXO or tight phimosis that failed to respond to a trial of topical steroid followed by preputial adhesiolysis or recurrence) undergoing circumcision were included in the study. The exclusion criterion was circumcision for ritual and urological indications in normal prepuce.

Ethical clearance

Approval for this study was obtained from the Institutional Ethical Review Board (vide Approval Letter No. SJMCH/IEC-257/2024).

## Results

**Figure 1:**
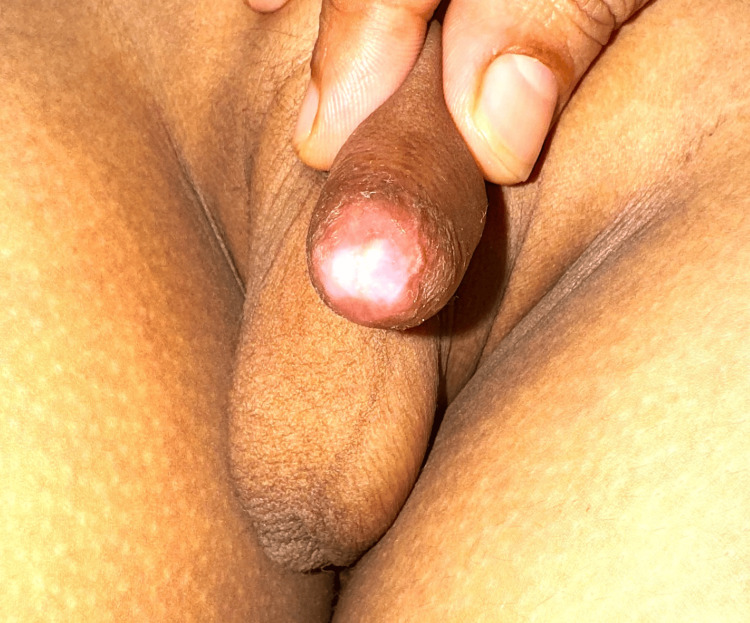
Balanitis xerotica obliterans: the glans is thickened with white depigmentation

During the study period, a total of 426 patients with phimosis presented to the Outpatient Department of Pediatric Surgery, of which 360 patients were successfully managed with conventional treatment with a brief course of steroid application followed by preputial adhesiolysis and 36 patients defaulted for follow-up. A total of 108 patients underwent circumcision during this period, of which 78 patients who underwent circumcision for ritual or urological indications with normal prepuce were excluded and 30 patients with PP were included (Figure 1).

The mean age of presentation was 9.6 (range: 4-16) years (one patient aged less than five years of age had meatal involvement with segmental vitiligo). The mean duration of symptoms was 84.5 (range: 7-400) days. Recurrence of PP was seen in six (20%) patients after a course of initial steroid application with preputial adhesiolysis. The symptomatology of children is highlighted in Tables 1, 2.

**Table 1:** Demographic data *Managed with a trial of topical steroid therapy with preputial adhesiolysis as per the institutional protocol

Clinical Characteristics	Number (Range)
Mean age at presentation	9.6 (4-16) years
Mean duration of symptoms	84.5 (7-400) days
Total patients with phimosis	426
Physiological phimosis*	360
Total circumcisions	108
Circumcision for ritual/urological indications	78
Circumcision for Pathological Phimosis	30

**Table 2:** Symptomatology of children with phimosis

Symptomatology	Number (Percentage)
Inability to retract prepuce	30 (100)
Ballooning of prepuce	20 (66.7)
Thin urinary stream	14 (46.7)
Preputial discoloration with plaques	9 (30)
Recurrent balanoposthitis	4 (13.3)
Acute balanitis	5 (16.7)

Inability to retract the prepuce and ballooning of the prepuce were the predominant complaints at presentation. Five children presenting with acute balanitis were treated with antibiotics prior to surgery. Meatal involvement of the urethra was observed in four (13%) patients, all of whom were managed with meatal calibration after circumcision (one patient needed meatotomy). All patients were treated with meatal calibration using topical steroids for six months, with documented normal uroflowmetry values by the three-month follow-up. Of the 30 cases in the study, biopsies of preputial skin were available for review in 25 patients, the findings of which are detailed in Table 3.

**Table 3:** Histologic characterization of circumcised preputial specimens in pathological phimosis

Histopathological feature	Biopsies (n=25, 83%)	Final histopathologic diagnosis		
		Lichen sclerosis (n=18, 72%)	Lichenoid dermatitis (n=4, 16%)	Chronic inflammation (n=3, 12%)
Epidermis				
Normal	13 (52)	8	3	2
Denuded/ulcerated	3 (12)	1	1	1
Atrophic	8 (32)	8	0	0
Acanthotic	1 (4)	1	0	0
Inflammation in dermis (predominant pattern)				
Lichenoid lymphocytic	22 (88)	18	4	0
Perivascular lymphocytic	3 (12)	0	0	3
Changes in interface dermatitis				
Necrotic keratinocytes	22 (88)	18	4	0
Basal vacuolar alteration	6 (24)	6	0	0
Prominent melanophages	3 (12)	1	2	0
Homogenization of papillary dermal collagen				
Present	18 (72)	18	0	0
Absent	7 (28)	0	4	3

**Figure 2:**
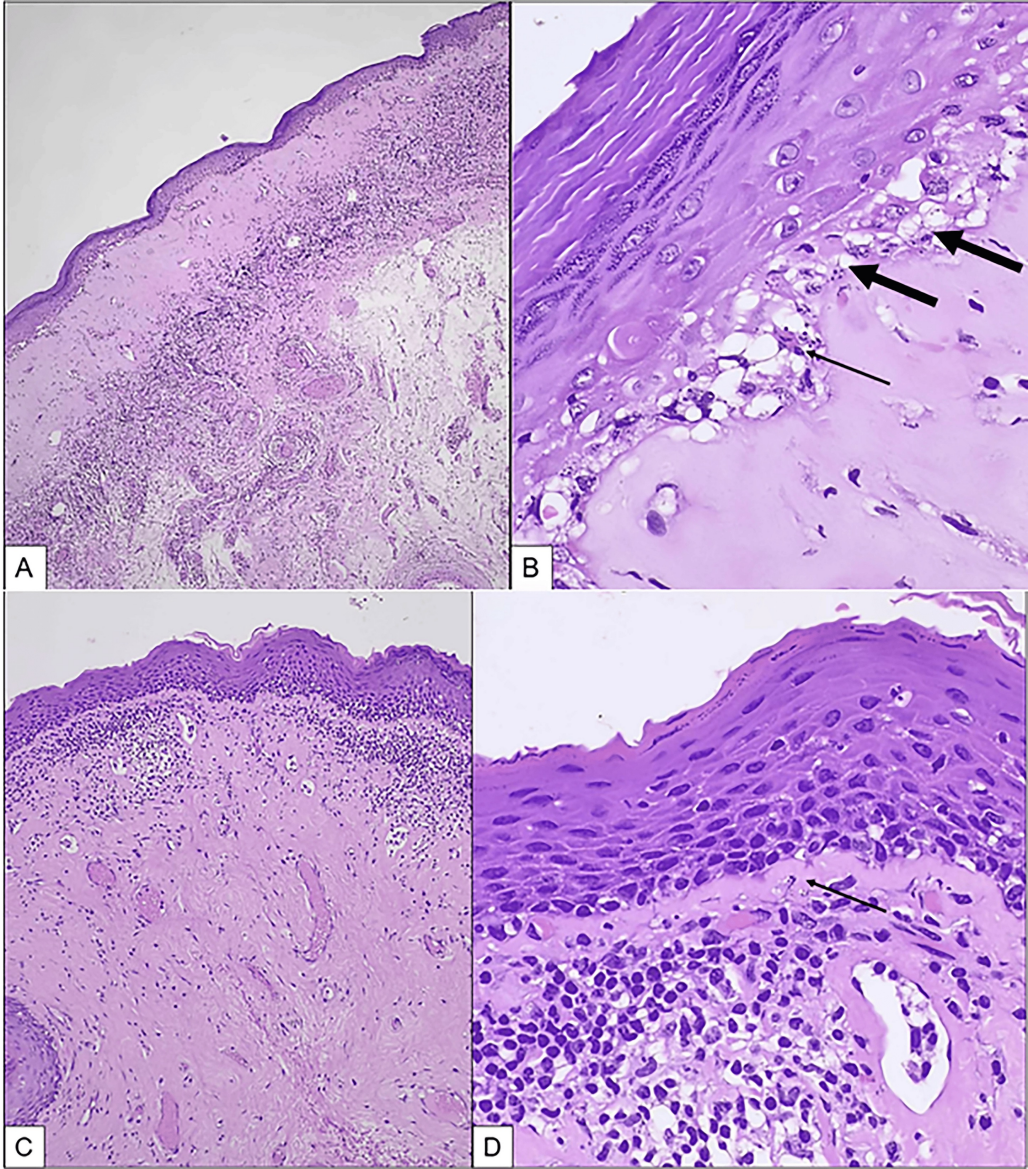
Preputial skin showing Lichen sclerosus. A. Note the atrophic epidermis, homogenized papillary dermis with subjacent lichenoid lymphocytic infiltrate (H&E, 100x). B. Necrotic keratinocytes (thin arrow) and basal vacuolar alteration (thick arrow) (H&E, 200x). C, D. Note the thin band of homogenized papillary dermis (arrow in D) with subjacent patchy lichenoid lymphocytic infiltrate (H&E, 100x and 200x, respectively).

**Figure 3:**
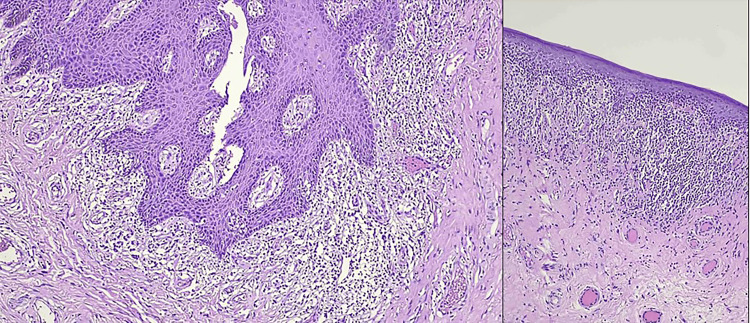
Preputial skin showing lichenoid inflammation. A, B. Note the lichenoid lymphocytic infiltrate and lack of homogenized papillary dermis (H&E, 200x and 100x, respectively).

Abnormal findings were noted in all cases of PP on biopsy; in five patients, biopsy was not sent or traceable. Lichen sclerosis (Figure 2) was seen in 18 (72%), lichenoid dermatitis (Figure 3) in four (16%), and chronic non-specific inflammation in three (12%) patients.

All patients with lichenoid changes were initiated on topical steroid applications (0.1% betamethasone/mometasone) as per institutional protocol, and monthly follow-up was initiated for a minimum period of six months, thereafter every six months for further six months.

## Discussion

This study highlights that relatively innocuous symptoms, such as the inability to retract the prepuce, ballooning of the foreskin, and recurrent preputial infections in children that have recurred or persisted with topical steroid therapy and preputial adhesiolysis, are likely to represent PP, with nearly all patients demonstrating lichenoid changes in the biopsy specimens. Circumcision, followed by long-term topical steroid application, is curative, with prompt resolution of symptoms even in those with meatal involvement, thereby reiterating the need for sustained close follow-up. This study also underscores the importance of routine histological examination of preputial specimens in PP. In our study, 72% of the cases (where biopsy was available) were diagnosed with lichen sclerosus. The epidermis was either normal or atrophic in a majority of cases. A rare case also had an acanthotic epidermis. This supports the view that the term “lichen sclerosus et atrophicus” is now obsolete as the epidermis is not always atrophic. While most cases of lichen sclerosus showed lichenoid lymphocytic inflammation, a perivascular mild lymphocytic infiltrate was seen in the few cases diagnosed as chronic inflammation. Homogenization of collagen is an essential feature to diagnose lichen sclerosus; thus, the lack of this feature in four cases was signed out as “lichenoid dermatitis.” These cases also showed changes in interface dermatitis such as necrotic keratinocytes and melanophages. These cases could probably portray early lichen sclerosus where sclerosus has not yet set in, thus emphasizing the importance of clinical correlation and histopathologic examination in such situations as early therapy can be beneficial [8].

The annual incidence of BXO as estimated from epidemiological studies is 3.01 and 0.322 cases per one thousand boys under the age groups of 15 years and 5 years, respectively [9]. The exact etiology of BXO is not clearly known, although various hypotheses including human papillomavirus, Epstein-Barr virus, Borrelia Burgdorferi, and auto-immune causation have been postulated [3,10,11]. Our Institutional Protocol for the management of most phimosis including physiological phimosis is a short course (three weeks) of topical steroid preparation, followed by preputial adhesiolysis [4]. However, refractory phimosis (unresponsive or recurrent) is usually a harbinger of BXO. Treatment of established BXO is a long course (six months in proven cases) of topical steroids such as mometasone (0.1%) or betamethasone (0.1%), although high potency steroids such as clobetasol, calcineurin inhibitors, and imiquimod may be needed in refractory, albeit rare, cases [12]. If untreated, these lesions may progress to catastrophic urethral/penile involvement with debilitating strictures [5]. Lichen sclerosus is potentially premalignant, and malignant transformation is reported in 4-8% of the cases after a prolonged latency of more than two to three decades [6].

Histologically, lichenoid changes may be differentiated into lichen sclerosus (classical) and lichenoid reaction. Histological changes include hyperkeratosis, parakeratosis, vacuolar degeneration with obliterative end arteritis, and variable lymphocytic infiltrate. The need for histological examination of prepuce arose following an initial report by Bochove-Overgaauw et al., who evaluated 135 preputial specimens of children with PP and noted that 27% of the biopsies detected BXO and that the clinical features were underestimated compared to histological findings in nearly 50% of the cases [13]. Naji et al. described their series of 112 boys who underwent circumcision in a normal prepuce for medical or religious indications and found a 15% incidental occurrence of BXO with lichenoid changes, thus underscoring the importance of routine biopsy evaluation of the excised prepuce [7]. Irkilata et al. reported a 10% incidence of incidental early lichenoid changes in preputial specimens of children undergoing routine circumcision [14]. Arena et al. demonstrated increased expression of CD8+ and CD57+ lymphocytes with overexpression of Ki67 and p53+ cells in preputial specimens of patients with juvenile lichen sclerosus, thereby confirming the proliferative nature and immune reaction [15]. Even in our study, we noted lichenoid changes in most specimens of PP and chronic non-specific inflammatory changes in the small minority of remaining cases. This reiterates the need for more robust, large-scale multicenter randomized controlled trials to validate the findings and suggests routine histological examination of all preputial specimens as a standard practice guideline.

Once diagnosed, definitive management is a long-term application of topical steroid cream. ElAgami et al. reported a statistically significant reduction (less than 8%, p=0.0165) in need for subsequent meatal dilatation in patients instituted on topical steroid therapy [16]. Circumcision with post-operative topical steroids is the advocated therapy in diagnosed lichen sclerosus for a minimum of 12 weeks, which is effective in most cases. In refractory cases, high-potency steroids such as clobetasol, intralesional triamcinolone, imiquimod, or calcineurin inhibitors may be given upto six months [17]. Regular post-operative follow is imperative; Snodgrass et al. reported a series of patients who were detected to have recurrent BXO, especially in a subset of those who underwent urethroplasty for hypospadias; despite initial therapy with high potency steroids and tacrolimus for >12 weeks at primary management post-surgery, recurrence was noted at mean interval of 26 months (range: 22-105 months), emphasizing the need for regular and long-term screening in such individuals [18].

Limitations

The limitations of this study are the retrospective study design and the lack of comparison of outcomes with the control group(circumcision for non-pathological conditions), causing variability in documentation and treatment compliance. The relatively small sample size may be explained by the institutional protocol, whereby only candidates with PP underwent circumcision. Our study followed up patients for a six-month period; however, considering the course of lichen sclerosus, a longer follow-up is preferable. Larger multicenter randomized controlled trials and detailed immunohistological analysis would be preferable to address the shortcomings of this study.

## Conclusions

Routine histological examination of preputial specimens in children with PP is effective in detecting early or established lichenoid changes. Prolonged treatment with topical steroid post-circumcision for at least six months is curative. Regular follow-up is essential to screen for urethral involvement.
